# Advances in neonatal cell therapies: Proceedings of the First Neonatal Cell Therapies Symposium (2022)

**DOI:** 10.1038/s41390-023-02707-x

**Published:** 2023-06-28

**Authors:** Atul Malhotra, Bernard Thebaud, Madison C. B. Paton, Bobbi Fleiss, Paris Papagianis, Elizabeth Baker, Laura Bennet, Tamara Yawno, Ngaire Elwood, Belinda Campbell, Kirat Chand, Lindsay Zhou, Tayla Penny, Timothy Nguyen, Salvatore Pepe, Alistair J. Gunn, Courtney A. McDonald

**Affiliations:** 1 Department of Paediatrics, Monash University, Melbourne, VIC Australia; 2 Monash Newborn, Monash Children’s Hospital, Melbourne, VIC Australia; 3 The Ritchie Centre, Hudson Institute of Medical Research, Melbourne, VIC Australia; 4 Regenerative Medicine Program, The Ottawa Hospital Research Institute (OHRI), Ottawa, ON Canada; 5 Department of Cellular and Molecular Medicine, University of Ottawa, Ottawa, ON Canada; 6 Neonatology, Department of Pediatrics, Children’s Hospital of Eastern Ontario (CHEO) and CHEO Research Institute, Ottawa, ON Canada; 7 Cerebral Palsy Alliance Research Institute; Speciality of Child and Adolescent Health, Sydney Medical School, Faculty of Medicine and Health, The University of Sydney, Sydney, NSW Australia; 8 RMIT University, Melbourne, VIC Australia; 9 Department of Pharmacology, Monash University, Melbourne, VIC Australia; 10 Royal Women’s Hospital, Melbourne, VIC Australia; 11 Department of Paediatrics, University of Melbourne, Melbourne, VIC Australia; 12 Departments of Physiology and Paediatrics, School of Medical Sciences, University of Auckland, Auckland, New Zealand; 13 Murdoch Children’s Research Institute, Melbourne, VIC Australia; 14 Perinatal Research Centre, University of Queensland, Brisbane, QLD Australia; 15 Department of Obstetrics and Gynaecology, Monash University, Melbourne, VIC Australia

## Abstract

**Abstract:**

Despite considerable advances, there is a need to improve the outcomes of newborn infants, especially related to prematurity, encephalopathy and other conditions. In principle, cell therapies have the potential to protect, repair, or sometimes regenerate vital tissues; and improve or sustain organ function. In this review, we present highlights from the First Neonatal Cell Therapies Symposium (2022). Cells tested in preclinical and clinical studies include mesenchymal stromal cells from various sources, umbilical cord blood and cord tissue derived cells, and placental tissue and membrane derived cells. Overall, most preclinical studies suggest potential for benefit, but many of the cells tested were not adequately defined, and the optimal cell type, timing, frequency, cell dose or the most effective protocols for the targeted conditions is not known. There is as yet no clinical evidence for benefit, but several early phase clinical trials are now assessing safety in newborn babies. We discuss parental perspectives on their involvement in these trials, and lessons learnt from previous translational work of promising neonatal therapies. Finally, we make a call to the many research groups around the world working in this exciting yet complex field, to work together to make substantial and timely progress to address the knowledge gaps and move the field forward.

**Impact:**

- Survival of preterm and sick newborn infants is improving, but they continue to be at high risk of many systemic and organ-specific complications.
- Cell therapies show promising results in preclinical models of various neonatal conditions and early phase clinical trials have been completed or underway.
- Progress on the potential utility of cell therapies for neonatal conditions, parental perspectives and translational aspects are discussed in this paper.

## Introduction

Research in neonatal cell therapies has been gathering momentum over the last decade. Multipotent (“stem”) cells are specialized cells defined by their ability to renew themselves, and potential to differentiate into different cell lineages. Not all cell therapies used in regenerative medicine are true stem cells, and hence the term “cell therapies” is more appropriate to refer to cells obtained from biological tissues that have the potential to repair, protect, and in some cases regenerate vital body tissues.

**Fig. 1:**
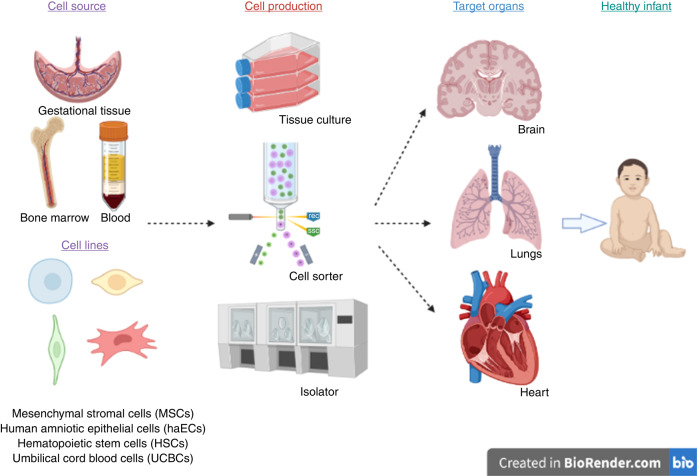
Neonatal cell therapies in current clinical trials. Infographic showing the cell sources, cell lines, cell production, and organs targeted in current neonatal cell therapy trials.

With advances in neonatal medicine, the survival of preterm and sick term newborn infants has improved substantially. Importantly, rates of preterm complications like chronic lung disease, brain injury and pulmonary hypertension remain high, as do the risks of long-term neurodevelopmental, pulmonary, cardiac, and metabolic complications are still substantial. Hence, it is imperative to explore other therapeutic alternatives. Cell therapies offer a potential new frontier (Fig. 1).

Cell therapies can be sourced from many biological tissues and can be broadly divided into gestational tissue derived or adult tissue derived cells. The primary cell types from gestational tissues are sourced from embryonic structures, cord blood, cord tissue, and placental tissue and membranes. The main sources of adult cell types include bone marrow, bone, skin and adipose tissue. Another source is induced pluripotent stem cells that have been reprogrammed from skin or other tissue types into an embryonic state.

Overall, most, but not all, preclinical studies suggest potential for benefit. The participants in this symposium all emphasized that many of the cells tested were not adequately defined, and we do not know the optimal cell type or starting time or numbers of cells or the most effective protocols for the targeted conditions, as discussed in the individual sections below. Critically, there is as yet no strong clinical evidence for benefit, although several early phase clinical trials are now assessing safety in newborn babies. In this review, we focus on the most prominent cell types that are being evaluated in pre-clinical and clinical translational studies for neonatal morbidities. We discuss mesenchymal stromal cells (MSCs), umbilical cord blood (UCB), and cord tissue-derived cells, and placental tissue and membrane-derived cells. Parental perspectives on this approach, and the lessons learnt from previous translational work of other neonatal therapies are considered.

## MSC therapies

MSC therapies have been investigated pre-clinically and clinically for decades across a range of conditions, especially those with an inflammatory basis. Many but not all preclinical studies have demonstrated benefit from MSCs in neonatal disorders.^1–14^ Clinically, MSCs show excellent patient safety profiles and apparently positive patient outcomes.^15^ The major issue at present is lack of definitive evidence for efficacy from randomized controlled trials. Importantly, MSCs can be readily obtained from a range of sources and the field has seen significant commercialization, with many off-the-shelf MSC products developed to offer long-term scalable solutions. Most preclinical and clinical neonatal research to date has focused on MSC therapy for improving lung and brain outcomes. However, there are limited studies investigating their utility for treating preterm brain injury.

### Updates on MSC treatment for perinatal lung injury

Progress in perinatal care has pushed the limits of viability of extreme preterm infants to around 22 weeks gestation. For the first time in decades of progress, neonatology is now confronted with the biological limits of viability.^16^ In part, this may explain the difficulty in decreasing the incidence/severity of bronchopulmonary dysplasia (BPD), the chronic lung disease of prematurity.^17,18^ It is important to appreciate that extremely preterm infants are born at the late canalicular stage of lung development, just when gas-exchange becomes possible with currently available supportive therapies.^19^ Thus, the therapeutic challenge is to allow lung growth while preventing injury of the infant’s extremely fragile lung.^20,21^

Exciting discoveries in regenerative medicine point towards potential effective interventions to prevent organ injury and/or restore function. MSCs are the most extensively studied cell type because of their ease of isolation, putative pleiotropic effects and safety profile.^22^ The discovery of the umbilical cord tissue and cord blood as a clinically relevant source of MSCs further positioned these cells as an ideal therapeutic approach for preventing complications of extreme prematurity. Extensive preclinical testing in rodent models of neonatal lung injury have established the lung protective properties of MSCs demonstrating the capacity of intravenous (IV) or airway delivered MSCs to improve lung histology, function and inflammation as well as attenuate pulmonary hypertension.^23,24^ The main mechanism of action in rodents seems to be immune modulation, but other effects have been ascribed to MSCs including pro-angiogenic, anti-oxidant, anti-fibrotic, and pro-lung growth.^25^ Interestingly, MSCs do not engraft and therapeutic benefit of MSCs is mediated via the release of extra-cellular vesicles (EVs), adding the possibility of a cell-free therapeutic approach.^26^

Of potential concern, the one study in non-human primates born at the limit of viability and given a single, intravenous dose of ten million human umbilical cord tissue-derived MSC per kg or placebo immediately after birth found no benefit for lung function after 2 weeks recovery.^7^ These contrasting data may reflect the model (extremely immature baboons compared to term born rodents), the use of a prophylactic infusion before inflammation was established, or simply the use of a single infusion, instead of repeated infusions over time. Large animal studies can be relatively difficult and expensive but as this study shows, they can be essential to help develop effective protocols.

The exciting findings in rodents have led to early phase clinical trials within than 10 years of the first reports from neonatal laboratory investigations. The available clinical evidence confirms a favorable safety profile of intra-tracheal administered cord blood-derived MSCs in extremely preterm infants.^27–29^ No therapeutic benefit has yet been reported, based on a single phase II trial.^30^ However, numerous early phase clinical trials are ongoing and will further inform us about the safety and potential efficacy of different sources of MSCs, different route of administrations, dosing and timing as well as patient populations most likely to benefit.^31^

Less encouragingly, the results of adult clinical MSC trials have been negative so far and do not match the promising preclinical studies,^32,33^ underscoring large knowledge gaps in our fundamental understanding of MSC biology. A likely key issue is imprecise characterization of cell type, leading to major controversy around MSCs and a critical request to “clear up the stem cell mess.”^34,35^ This is justified based on a recent scoping review demonstrating the lack of clarity in defining MSCs and reporting results in the preclinical and clinical MSC literature, despite the existence of minimal criteria from the International Stem Cell Society (ISCT).^35^ A Delphi survey is underway to establish some consensus on definition of MSC and guidelines for clinical trial reporting to enhance transparency and quality of interpretation of MSC trials. Novel, single cell technology now allows in depth characterization of MSC and paired with functional studies, may enable progress towards harnessing the putative repair potential of these cells^36^ or their by-products.

Interestingly, there is increasing evidence that EVs released by MSCs exert robust lung protective effects in experimental hyperoxia-induced neonatal lung injury.^37^ EVs are 40–100 nm in size and represent a specific subtype of secreted membrane vesicles formed through the fusion of multivesicular endosomes with the cell membrane.^38^ Previously regarded as the garbage bin of cells, EVs are an important form of cell-to-cell communication. EVs transfer proteins and genetic information via mRNA, and miRNA. Their rich cargo makes EVs intriguing therapeutic vehicles for complex diseases such as BPD, potentially with less risk of off-target effects. Willis et al. for example, demonstrate the capacity of MSC-derived EVs to polarize macrophages from a pro-inflammatory M1- to a pro-repair M2 phenotype.^26^ Subsequent studies confirmed the potent immunosuppressive activity of EVs through phenotypically and epigenetically reprogramming monocytes.^6^

Well conducted animal studies adhering to ARRIVE guidelines and assessing side-by-side efficacy, dosing timing and route of administration will provide stronger pre-clinical evidence and rationale for cell or cell-free therapies to ensure success of clinical translation. In parallel, manufacturing is an entire research field in itself. Collaboration with bioengineers will be critical to create the safest and most effective cell product. As such, this Neonatal Cell Therapies Symposium was a welcome opportunity to advance our knowledge in this burgeoning field of regenerative medicine specifically dedicated to this patient population.

### Updates on clinical trials using MSC treatment for perinatal brain injury

Clinical trials using MSCs to improve neonatal brain outcomes have included for treatment of congenital heart conditions,^39^ hypoxic–ischemic encephalopathy,^40,41^ and neonatal/perinatal stroke.^42,43^ Most phase I research has focused on intraventricular hemorrhage and perinatal arterial ischemic stroke.^42,43^ Collectively, these dose-escalation studies show feasible administration of MSC within 28 days of life and further demonstrated that MSCs are safe and well tolerated, priming the field for phase II trials.

Overall, the intraventricular and intranasal routes have been favored along with single doses of MSCs in the acute or sub-acute phase of injury. However, administration of repeated dosing into the tertiary phase is strongly supported by preclinical literature, as well as other treatment routes, and the use of MSC products (exosomes, EVs).^44–46^ Future neonatal brain research of MSC treatments in clinical trials may need to explore these options.

### Common advances in MSC research for improving neonatal outcomes

We commonly see calls for research to address and improve on the considerable heterogeneity in MSC source, specific cell type selected and treatment regimen (including dose, repeat dose, route, and timing). But perhaps the better call is not for standardization; it should be for researchers to better define MSC products from the start. Overall, we should consider that not every MSC needs to be equal, using the current limited panel of markers, to be effective. Parallel investigations are warranted.

As mentioned above, manufacturing is critical, as each step in the process of making a repair cell will affect its function, i.e., therapeutic effect and thus trial outcome. For example, current practice is to collect MSCs from umbilical cords from healthy term babies. An intriguing question is whether autologous therapy is a viable alternative. However, little is known about the effect of perinatal conditions accompanying preterm birth such as preeclampsia or chorioamnionitis on MSC function.

Commercial MSC products have been used in clinical trials, which may offer some added benefit and ease. The emphasis should now be on determining why one particular type of commercial MSC product is efficacious, how efficacious/potent they are in particular indications, how they should be applied, and when they should be applied so research efforts can be informed and focused.

Promisingly, we are seeing these principles being used. For example, the European union funded the PREMSTEM consortium (www.premstem.eu), in which standardized, scalable, and commercially produced MSCs are being comprehensively tested through in vivo and in vitro screening as a therapy for encephalopathy of prematurity. This includes testing MSC product in two small animal models at different doses, time points and administration routes. This will be followed by validation across further small and large animal models using clinically relevant outcome markers. This type of detailed, labor-intensive standardized screening is one that the PREMSTEM team, built of clinical and pre-clinical researchers, believes will help close the translational gap.

## Cord blood-derived cell therapies

Human umbilical cord blood (UCB) has been identified as a heterogeneous source of cells with potential anti-inflammatory, neuroprotective and regenerative properties including MSCs, hematopoietic stem cells (HSCs), endothelial progenitor cells (EPCs), monocytes, B cells, and T regulatory cells.^47–49^ Due to these properties, there has been increasing research into the use of UCB-derived cell therapies for the treatment of a wide-range of neonatal conditions including neurological, respiratory, and cardiovascular pathologies. Currently, most of this research has been conducted in pre-clinical models,^50–54^ but early phase clinical trials involving human neonates are in progress.

A systematic meta-analysis of 55 studies demonstrated that UCB-derived cell therapy was an efficacious treatment in pre-clinical models of perinatal brain injury.^55^ UCB-derived cells were thought to exert their neuroprotective effects by significantly reducing microglial activation, astrogliosis, apoptosis, neuroinflammation and so reducing infarct size, and improving numbers of neurons and oligodendrocytes and overall motor function. Further analysis suggested that these therapies may be more efficacious in IVH models compared to models of hypoxia ischemia and when given through local routes as compared to systemic routes.^13^ However, given the heterogeneity of design of these pre-clinical studies, including the route and timing of administration, cell dosage, number of doses, and UCB cell type, the overall certainty of evidence was low. Penny et al. have shown, for example, that UCB improved outcomes in both male and female rats after neonatal hypoxic-ischemic brain injury,^56^ and that multiple doses of UCB were more effective than a single dose.^57^

### Updates on clinical trials of UCB derived cell therapies in neonates

Based on the pre-clinical evidence supporting UCB-derived cell therapy for many neonatal conditions, there have been 12 completed clinical studies to date (mostly phase I/II studies) exploring UCB or UC tissue-derived cell therapy, with most common neonatal diseases studied being HIE (33%) and bronchopulmonary dysplasia (25%).^58^ Additionally, there are a further 24 newborns trials are currently in progress. Recently, UCB derived cell therapies have been trialed for the first time in extremely preterm infants, showing promising feasibility.^59^ Safety trials in this population are ongoing.^60^ An important consideration is the issue of altered immune cell development in preterm infants, and the possibility that poor immune function may affect the efficacy of these cells. This again emphasizes the need to fully characterize the cells that are being administered.

### Updates on clinical trials of UCB-derived cell therapies for neonatal heart conditions

For children born with congenital heart disease, hypoplastic left heart syndrome (HLHS) without surgical intervention can lead to rapid heart failure and death after birth.^61^ HLHS is a complex multi-factorial combination of developmental conditions leading to absent or underdeveloped left ventricle, mitral valve, aortic valve, and narrow ascending aorta, each with varied extent of abnormal morphology and dysfunction.^62^ Initial survival requires staged cardiac surgical palliation that establishes a viable systemic circulation supported by the right ventricle (in the absence of a functional left ventricle), beginning with the Norwood operation within the first few days of life, a second-stage operation at 3-4 months of age and the Fontan operation within 3–5 years.^62^ While there have been recent efforts to treat children with HLHS using UCB-derived cells, adjunct cell therapy has only been performed in older, more stable infants at the second-stage operation.^63^ As the interstage period following the Norwood operation features very high morbidity and mortality, earlier UCB-cell therapy is proposed to target this highly vulnerable period by stimulating positive cardiac remodeling and preservation of ventricular function,^53^ A recently completed clinical trial^61^ currently in final analysis, aimed to assess the safety and feasibility of a novel method of autologous UCB-cell delivery directly to the coronary vasculature during the Norwood operation in neonates with HLHS at day 2–3 of life. This may not be feasible in all infants, but the results will be of great interest.

### Regulatory considerations

As we progress UCB-derived cell therapy as a potential future treatment for neonatal conditions, it is important to acknowledge and explore the regulatory considerations surrounding the usage of cord blood as well. Cord blood banks will undoubtedly play a key role in cellular therapies, as they will be a key source of donor cells for allogeneic/non-homologous hematopoietic stem cell transplantation (HSCT). Key considerations include ensuring a robust quality framework is implemented, and that protocols are put in place for donor consent and ethical review of non-homologous use of cord blood. These steps will help further progress the translation of UCB cell therapies to human neonates.

## Placental tissue stem cells

The healthy human placenta is made up of a fetal side (chorion) and a maternal side (basal), which are held together by anchoring villi. On the fetal side of the placenta, membranes called the chorion and the amnion harbor stem cells. The chorion contains rare chorionic MSCs and amniotic membrane and fluid contains amniotic epithelial cells (AECs). Human amnion epithelial cells (hAECs) have been used pre-clinically^64,65^ and in early phase clinical trials to treat bronchopulmonary dysplasia^66,67^ and hypoxic–ischemic (HI) brain injury.^68^ More work is needed to determine the effective dose, timing, frequency and number of doses for hAEC administration in all disease states.

### Updates on hAEC-derived cell therapies for neonatal brain injury

After HI, perinatal brain injury evolves, allowing distinct windows of opportunity for neuroprotection and neurorepair.^69^ hAECs modulate injury through anti-inflammatory and anti-apoptotic effects, stabilization of cellular metabolism, and by promoting cell proliferation, maturation and differentiation.^70^

The optimal timing of hAEC dosing for HI injury is yet to be established. The efficacy of hAECs in HI injury was assessed in fetal sheep models of preterm HI injury to examine this question.^71–73^ Preterm animals were given a single intracerebroventricular hAEC infusion (1 × 10^6^), at either 2 or 24 h post-HI injury induced by umbilical cord occlusion. The histological effects at 1 week after injury were similar after both dosing time points.^71^ Compared with controls, oligodendrocytes survival was not improved by hAEC administration at either time point, but interestingly, greater thalamic and striatal protection was seen after infusion at 24 h. It is critical to appreciate though that multiple doses may have offered improved neuroprotection.^74^

In preterm fetal rodent models of HI injury, an intranasal infusion of hAECs (5 × 10^6^) administered at 24 h, 3 and 10 days, and assessed histologically at 21 days after HI, was associated with significant neuroprotection. Oligodendrocyte maturation and myelination was restored, brain weight increased, microglial and astrocyte number were reduced, and improved functional development of the brain were observed.^72^ In contrast to these studies, intravenous hAECs given 2 hours post insult did not confer neuroprotection or anti-inflammatory effects in term rodent models of perinatal HI injury.^73^ Neuroprotection failure may relate to age, route of administration, or nature of the insult, but these data raise the intriguing possibility that early cell treatment may not be optimal.

### Updates on hAEC-derived cell therapies for BPD

Multiple animal models of BPD have shown that hAECs can restore lung architecture and improve lung function. For example, in one study, preterm lambs were exposed to antenatal LPS and then delivered, resuscitated and provided with respiratory support for the first 7 days of life.^75^ Respiratory support replicated current neonatal practice beginning with mechanical ventilation, and aiming to wean to continuous positive airway pressure (CPAP) and room air. Within the first hours of life, hAECs (30 × 10^6^ cells) or saline were administered IV. The respiratory support required by lambs exposed to antenatal LPS was not changed by hAEC treatment. However, lambs who received hAEC tended to have lesser histological lung injury and inflammation. This may indicate some capacity for hAECs to repair injured lung tissue without significantly impacting gas exchange or requirements for respiratory support at day 7 of life.

Again, the optimal timing of dose administration requires investigation. Rodent models of BPD suggest that administration earlier in the course of injury confers greater benefit.^76^ This finding was replicated in lamb models of BPD (Papagianis, unpublished data). hAECs were more effective at reducing lung inflammation and injury when delivered on day 1 of life compared with day 3. Early treatment with hAECs appears to be important for greatest therapeutic effect.^76^ However, preterm studies have also demonstrated the efficacy and benefits of delayed hAEC treatment in a mouse model of BPD.^65^ Significant further work is now required to standardize studies to systematically optimize doses, dosing regime and routes relative to age, insult type(s).^70^

While there are important questions to address, this preclinical evidence demonstrates that hAEC are able to protect and restore lung architecture in BPD models via immune modulation, pro-angiogenic effects and activation of the stem cell niche. As preclinical work to refine aspects of our understanding of hAEC therapy progresses, it is important to determine if indeed hAECs are tolerated by preterm infants. Unlike other cell therapies, hAECS don’t have an extensive safety profile. Indeed the first in human study was conducted in infants with bronchopulmonary dysplasia.^66,67^ A dose escalation study is now underway recruiting extremely preterm infants at high risk of BPD during early weeks of life.^77^

### Next steps for placental tissue-derived stem cell therapies

There has been limited research into combination cell therapies to treat neonatal conditions. Chand and colleagues examined the effects of combined placental derived MSCs and endothelial colony-forming cells (ECFC—an EPC) in a piglet model of fetal growth restriction.^78^ This combination, termed cECFC, increased vessel density without evidence of excessive angiogenesis, as well as enhancing blood brain barrier integrity and modulating anti-inflammatory pathways in the brain. Treatment with MSCs alone did not exert these beneficial effects on vasculature. It is not yet clear whether these cells participate in off-target engraftment events, such as in the lungs and other organs nor whether this treatment demonstrates long-term efficacy. Nevertheless, combined MSC and ECFC therapy may have potential to promote neuroprotection in neonates via targeting of both inflammation and the NVU.

A further area for consideration is the use of a combined epithelial stem cell and stromal cell therapy. To our knowledge this has not yet been studied but has merit, as both epithelial and stromal cells are essential to normal development, regeneration and wound healing in healthy individuals. The major concern is the capacity to form teratomas when delivered as a combined therapy. The evaluation of a combined product must include detailed hAEC: MSC ratio studies, along with timing, dose regime and route evaluation to assess therapeutic efficacy, and long-term follow-up to identify any teratomas.

Recent research has shown that like MSCs, hAECs shed biologically active nanoparticles (EVs) that exert therapeutic effects in some settings of inflammation and tissue damage.^79^ They can cross the blood brain barrier and consist of a lipid bilayer with incorporated transmembrane proteins, and a hydrophilic core containing cargo such as mRNA, miRNA, proteins and signaling molecules.^80,81^ hAEC-EVs increase macrophage phagocytosis, reduce neutrophil myeloperoxidases, and directly suppress T cell proliferation thereby improving tissue repair in the lung.^79^ We believe that the same mechanisms of action will be beneficial in the setting of preterm brain injury. Pragmatically, hAEC-EVs have advantages compared to hAEC therapy because they are more easily/widely translatable into clinical care, they can be provided as an off-the-shelf therapeutic without the expense of complex cold chain logistics. EVs can be mass-produced in a fashion similar to traditional pharmaceuticals, and thus offer a more stable form of regenerative medicine that will be both more readily available and more affordable than whole cell therapies.

### Parental perspectives

When a parent or prospective parent of a preterm or sick newborn baby is asked to participate in any research, it is a unique circumstance—unlikely any other field of medicine—of consenting to research of a person who may not be born yet, has never been home or spent time as a healthy individual. When parents are asked for consent to co-opt their child into a research study, which is experimental at best, even world first, or first-in-human in some cases, the complexities of this process cannot be overstated. While cell therapies feature high in the suite of upcoming, promising experimental therapies for most neonatal conditions,^82,83^ most of the clinical trials are at a safety or feasibility stage.^30,43,60,66,84^

One co-author/parent (B.C.), offered several salient points after participating in an early phase cell therapy research are highlighted here. The most important factor is a feeling of trust with the treating clinical team, to assist them in trying to improve care of not just “my” baby but similar babies in the future, the altruistic nature of such an exercise, and uncertainty associated with any safety study. Involving parents and other consumer representatives early in the design of such cell therapy studies is critical and the partnership with investigators, scientists, hospitals and hospital ethics committees is one way to guarantee fair representation and balanced views on this vital stage of translational research of these therapies.^85,86^

### Translational considerations

Learning from past experiences of research translation is critical. The successful translation of therapeutic hypothermia for hypoxic–ischemic encephalopathy in term and near-term infants, from structured animal studies,^87^ through small randomized pilot studies,^88^ to large randomized controlled trials,^89^ to become routine practice in high income countries, was the validation of previous decades of research. In retrospect, the key features are highlighted here. First, preliminary studies established that it was possible to recruit a very high risk (greater than 50% risk of death or disability) term and near term infants with moderate to severe encephalopathy, based on need for resuscitation, presence of perinatal acidosis, and bedside examination using the clinical criteria.^90^ In turn, the animal studies closely replicated key features of acute perinatal HI and evolving encephalopathy, including delayed onset of seizures and failure of oxidative metabolism.^91^ Critically, in large animal models, induced hypothermia had a huge effective size, typically improving neuronal survival by two to six fold in most regions, often to near sham control values.^92,93^ This is vital, when we reflect that in meta-analysis of human trials, relative risk for outcomes was approximately reduced by ~15%.^89^

Finally, recent studies emphasize that proof of principle is not sufficient to consider successful clinical translation. Early initiation of treatment with erythropoietin or the noble gas xenon offered substantial neuroprotection in a wide range of models.^94^ By contrast, in the fetal sheep, delayed treatment within the first 6 h had limited or no benefit.^95–97^ In turn, in large, well powered RCTs found no benefit. The likely reason for the lack of effect is that treatment was started much later that was tested in animal studies. For example, in the HEAL trial, treatment with erythropoietin was started within 26 h after birth^98^ and the TOBY-Xe trial,^99^ Xenon was given at median of 10 h after birth (range 4–12.5 h). This experience strongly suggests that strong preclinical evidence with realistic protocols that are likely to be beneficial in humans is needed before significant translational studies are undertaken, and trial protocols should strive to avoid going outside their preclinical evidence base.

At present, the evidence discussed above supports proof of principle that cell therapy can be beneficial in multiple neonatal settings. What we need now is much more targeted information. The outcomes of preterm infants are highly heterogeneous. For example, although 5–15% of extremely preterm infants will develop cerebral palsy and around 30% some other type of developmental disorder. These change with every week of gestation and, increase in birth weight. Can monitoring and blood biomarkers identify the infants at highest risk of disability? Brain injury in preterm infants appears to be multifactorial, involving inflammation and hypoxia-ischemia, and can occur in the antenatal, perinatal and postnatal periods. If we can find ways to identify when infants can benefit from treatment that would make it far easier to define the window of opportunity. Finally, and not least, some protocols have had marginal benefit in animal studies and so are unlikely to translate. As reviewed above this is likely related to multiple factors such as type of injury, cell therapy, doses, frequency, and route of administration.

A further important consideration for translation are cell therapy infusion protocols. Early phase hAECs studies highlighted importance of this when it was discovered that infusion protocols delivered less than 20% of the intended cell dose.^100^ A series of bench top experiments described the dose distribution during the hAEC infusion and, using simple measures and readily available equipment, a reliable infusion protocol was designed. Lastly, state of the brain environment can have a dramatic impact on whether cell therapy may be beneficial or not. For example, one preclinical study found after HI in neonatal rats, therapeutic hypothermia reduced brain inflammation but had a deleterious effect for subsequent cell therapy, such that the combination was worse than either intervention alone.^101^

### Summary and future

This review highlights advances made in cell therapies for neonatal conditions. While some early phase trials have already been completed, further dose finding and efficacy trials for most of these therapies need to be conducted. In order to support successful translation, we propose agreement on standardized pre-clinical studies, controlled for type of insult, stage of maturation, timing of treatments after the injury, cell doses, routes and characterization of longer term outcomes. It is widely recognized that it is unlikely that one preclinical model is superior to others, and a variety of preclinical models are more likely to replicate the substantial variation in perinatal populations who may benefit from cell therapies. Different types of ‘stem’ or other cells can then be compared within these models. This evidence base will be pivotal to understand the timing of injury mechanisms and thus when best to treat, and whether the type of cell makes a substantive difference for particular organs, types of injury and of course individual babies. There is also a critical need for standardization of the definition (characteristics) of the cell therapies used to treat neonates, including ECVs.

Additionally, some unique challenges face the field of neonatal brain research. These include how to best measure and harmonies efficacy outcomes, particularly in the short term. In these instances, neurodevelopmental assessments administered as early as 3-months’ corrected age provide enormous predictive value of disability.^102^

Finally, all research groups with interest in this complex field of translational research need to regularly connect and collaborate to make meaningful advances to move the field forward.

## Data Availability

Data sharing is not applicable to this article as no datasets were generated or analyzed during the current study.
